# Increased interferon-γ levels and risk of severe malaria: a meta-analysis

**DOI:** 10.1038/s41598-022-21965-z

**Published:** 2022-11-07

**Authors:** Aongart Mahittikorn, Wanida Mala, Frederick Ramirez Masangkay, Kwuntida Uthaisar Kotepui, Polrat Wilairatana, Manas Kotepui

**Affiliations:** 1 Department of Protozoology, Faculty of Tropical Medicine, Mahidol University, Bangkok, Thailand; 2 Medical Technology, School of Allied Health Sciences, Walailak University, Tha Sala, Nakhon Si Thammarat, Thailand; 3 Department of Medical Technology, Faculty of Pharmacy, University of Santo Tomas, Manila, Philippines; 4 Department of Clinical Tropical Medicine, Faculty of Tropical Medicine, Mahidol University, Bangkok, Thailand

**Keywords:** Immunology, Diseases

## Abstract

Interferon (IFN)-γ contributes to the pathogenesis of severe malaria; however, its mechanism remains unclear. Herein, differences in IFN-γ levels between patients with severe and uncomplicated malaria were evaluated using qualitative and quantitative (meta-analysis) approaches. The systematic review protocol was registered at PROSPERO (ID: CRD42022315213). The searches for relevant studies were performed in five databases, including PubMed, Scopus, Embase, MEDLINE and Web of Science, between 1 January and 10 July 2022. A meta-analysis was conducted to pool the mean difference (MD) of IFN-γ levels between patients with severe malaria and those with uncomplicated malaria using a random-effects model (DerSimonian and Laird method). Overall, qualitative synthesis indicated that most studies (14, 58.3%) reported no statistically significant difference in IFN-γ levels between patients with severe malaria and those with uncomplicated malaria. Meanwhile, remaining studies (9, 37.5%) reported that IFN-γ levels were significantly higher in patients with severe malaria than those in patients with uncomplicated malaria. Only one study (4.17%) reported that IFN-γ levels were significantly lower in patients with severe malaria than those in patients with uncomplicated malaria. The meta-analysis results indicated that patients with severe malaria had higher mean IFN-γ levels than those with uncomplicated malaria (*p* < 0.001, MD: 13.63 pg/mL, 95% confidence interval: 6.98–20.29 pg/mL, I^2^: 99.02%, 14 studies/15 study sites, 652 severe cases/1096 uncomplicated cases). In summary, patients with severe malaria exhibited higher IFN-γ levels than those with uncomplicated malaria, although the heterogeneity of the outcomes is yet to be elucidated. To confirm whether alteration in IFN-γ levels of patients with malaria may indicate disease severity and/or poor prognosis, further studies are warranted.

## Introduction

Malaria is one of the leading causes of death worldwide, with an estimated 627,000 malaria deaths reported in 2020^1^. The majority of these deaths were reported in children under 5 years of age in six countries of Africa, including Nigeria (27%), the Democratic Republic of the Congo (12%), Uganda (5%), Mozambique (4%), Angola (3%) and Burkina Faso (3%)^1^. Most malaria deaths were caused by *Plasmodium falciparum* infection; whereas, a minority were caused by other *Plasmodium* spp.^2–5^.

Immune responses to malaria have been described previously^6–8^. During malaria infection, pro- and anti-inflammatory cytokines play a role in protection against infection or disease pathogenesis^9^. Pro-inflammatory cytokines are produced by leukocytes such as neutrophils, lymphocytes and monocytes or other cells such as macrophages, endothelial cells, fibroblasts and mast cells^10–14^. These pro-inflammatory cytokines are interleukin (IL)-1, tumour necrosis factor (TNF)-α, interferon (IFN)-γ, IL-12 and IL-18^15,16^. IFN-γ is one of the pro-inflammatory T helper 1 (Th1) cytokines involved in protection against malaria and parasite clearance^9^. In combination with TNF-α and IL-12, IFN-γ inhibits parasite growth, stimulates phagocytosis and enhances the clearance of parasitised erythrocytes^17^.

Previous studies have indicated that elevated levels of pro-inflammatory cytokines, including IFN-γ, contribute to the severity of cerebral malaria^18,19^ and severe anaemia^17,20^, while also being associated with an increased likelihood of death^21,22^. Nevertheless, the role of IFN-γ in malaria severity remains controversial, and previous studies have included only a small number of participants with severe malaria^23–26^. To date, no meta-analysis has been used to unveil differences in IFN-γ levels among patients with severe and uncomplicated malaria. Therefore, in this study, the differences in IFN-γ levels between patients with severe malaria and uncomplicated malaria were estimated using a meta-analysis. These findings are essential to understanding the pathogenesis of malaria and guiding further studies.

## Materials and methods

### Protocol and registration

A systematic review and meta-analysis were conducted according to the recommendations of the Preferred Reporting Items for Systematic Reviews and Meta-Analyses (PRISMA) (S1 PRISMA Checklist)^27^. The systematic review protocol was registered at PROSPERO (ID: CRD42022315213).

### Definition of severe and uncomplicated malaria

Severe falciparum malaria is defined as the presence of *Plasmodium* parasitemia with one or more of the following complications: impaired consciousness, severe malarial anaemia, renal impairment, significant bleeding, acidosis, jaundice, prostration, multiple convulsions, shock, hypoglycaemia and hyperparasitemia. Severe vivax malaria is defined similarly to severe falciparum malaria, but there are no thresholds for parasite density^28^. Uncomplicated or mild malaria is the presence of *Plasmodium* parasitemia without the characteristics of severe malaria.

### Eligibility criteria

Studies reporting IFN-γ levels in patients with severe and uncomplicated malaria that met the PICO question criteria were included in this systematic review. The exclusion criteria were animal studies, in vitro studies, case reports or case series, review articles, studies with missing article information online, studies for which full-texts were unavailable, conference abstracts without full data, studies for which data of IFN-γ in both groups of patients could not be extracted and studies that reported IFN-γ levels after patients were treated.

### Search strategy

The search terms were chosen based on the Medical Subject Headings. A combination of search terms with Boolean operators was used as follows: ‘(interferon OR IFN OR interferon-gamma OR interferon-g OR IFN-g OR interferon-γ OR IFN-γ) AND (severe OR complicated) AND (malaria OR plasmodium)’. The searches were conducted in PubMed, Scopus, Embase, MEDLINE and Web of Science between 1 January and 10 July 2022 without a restriction on the publication date (Table S1). The searches were limited to English language only.

### Study selection

Two authors (MK and KUK) independently performed study selection. First, the titles and abstracts were screened for potentially relevant studies. In cases for which information from titles and abstracts was not sufficient for inclusion, the article was retained for full-text examination. Second, the full-texts of relevant studies were examined to find studies that met the eligibility criteria. Finally, disagreement between the two authors in study selection was resolved by discussion to form a consensus.

### Data extraction

Two authors (KUK and AM) carried out the data extraction, and data from the included studies were cross-checked by another author (MK). As a result, the following data were extracted to the spreadsheet: the name of the first author, publication year, study location, period of data collection, number of patients, age group, percentage of male participants, data on IFN-γ levels (qualitative data, mean with standard deviation or median with range for meta-analysis), parasite density, the technique used for detecting malaria parasites and technique used for measuring IFN-γ.

### Quality of the included studies

The methodological quality of the included studies was evaluated using the Strengthening the Reporting of Observational Studies in Epidemiology (STROBE) checklist for cross-sectional, prospective observational, and case–control studies^29^.

### Data syntheses

Data syntheses included qualitative and quantitative syntheses. A qualitative synthesis was the narrative synthesis of the difference in IFN-γ levels between patients with severe and uncomplicated malaria. A quantitative synthesis (meta-analysis) was conducted to pool the mean difference (MD) of IFN-γ levels between patients with severe and those with uncomplicated malaria. The random-effects model was employed to pool the MDs using the DerSimonian and Laird method^30^. The mean and standard deviation were estimated from the median and range as described previously^31^. Comparing the mean and standard deviation between groups of participants was carried out using a protocol described previously^32^. If the standard deviation was unavailable, a value was borrowed from other studies (with the lowest standard deviation) in the same meta-analysis^33^. The heterogeneity of the estimated effects between studies was assessed using Cochrane Q and I^2^ tests. A Cochrane Q test result with a significant value (p) less than 0.1 or an I^2^ result of more than 50% indicated heterogeneity in the estimates of effect between studies. When heterogeneity was revealed, a meta-regression analysis was performed to identify the source(s) of heterogeneity in the effect estimates. Then, a subgroup analysis was performed to determine the differences in the effect estimates between the subgroups of interest. Finally, a sensitivity analysis using the leave-one-out method^34^ was performed to test whether the meta-analysis was robust. Sensitivity analysis between studies that reported mean/standard deviation and median/range and studies that reported mean without standard deviation was conducted to assess the effect of changing the assumptions made. The sensitivity analysis between studies that reported mean/standard deviation and those with median/range of IFN-γ levels in patients with severe and uncomplicated malaria was conducted using a subgroup analysis. Publication bias was assessed by visually assessing funnel plot asymmetry and Egger’s test for small-study effects. If publication bias was found, the trim-and-fill method^35^ was applied to adjust the pooled effect estimates. The meta-analysis was performed using Stata version 17.0 (College Station, TX: StataCorp LLC, USA).

## Results

### Search results

**Figure 1:**
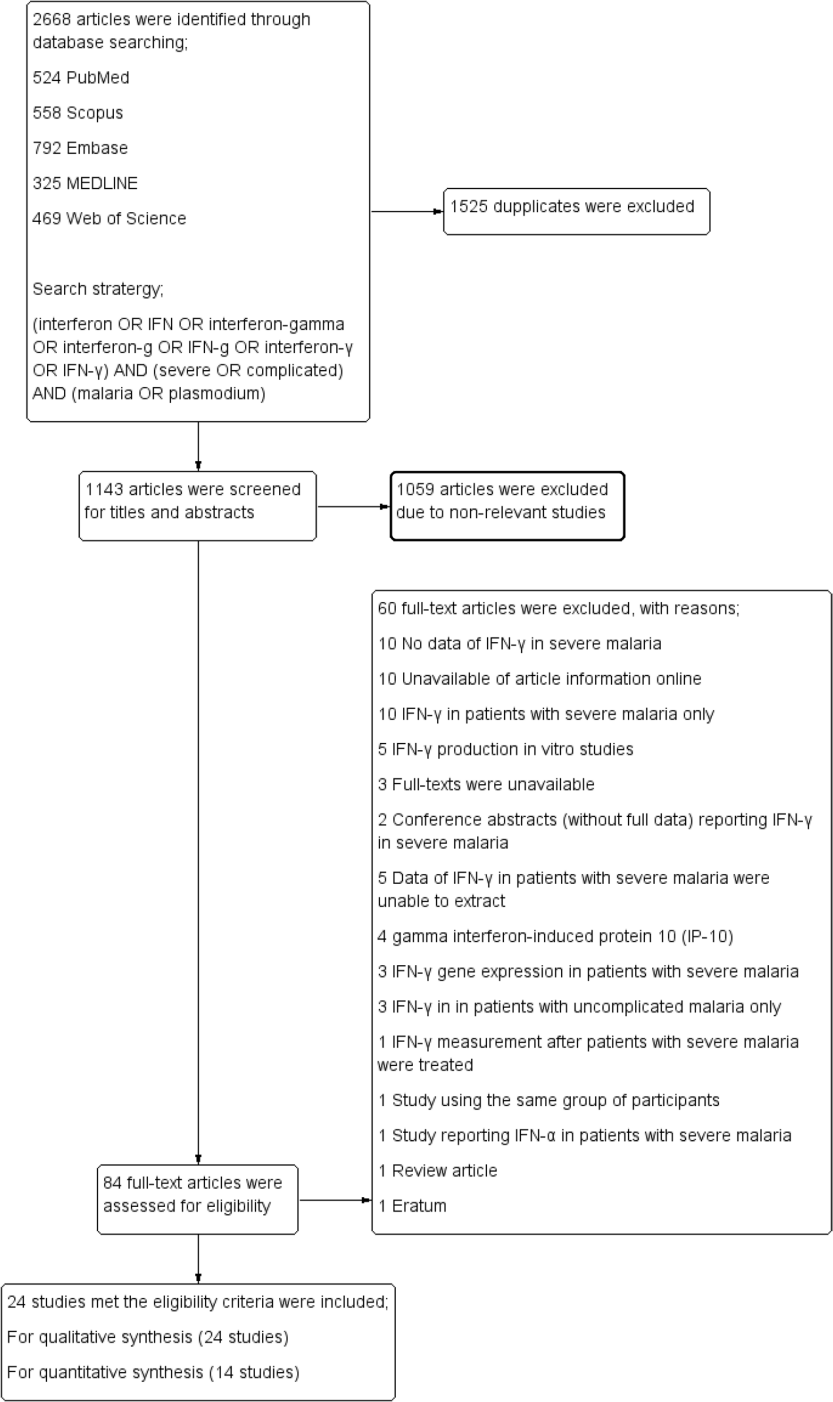
Study flow diagram.

A total of 2668 articles were identified from a database search, including 524 articles from PubMed, 558 from Scopus, 792 from Embase, 325 from MEDLINE and 469 from Web of Science. After study selection, 24 studies that met the eligibility criteria were included in the review (Fig. 1).

### Characteristics of the included studies

The included studies were published between 1990 and 2000 (3 studies, 12.5%), 2000 and 2010 (10 studies, 41.7%) and 2011 and 2017 (11 studies, 45.8%). The study designs were as follows: case–control (10 studies, 41.7%), prospective observational (9 studies, 37.5%), cross-sectional (3 studies, 12.5%) and prospective cohort (2 studies, 8.33%) studies. The studies were located in Africa (11 studies, 45.8%), Asia (7 studies, 29.2%), South America (3 studies, 12.5%), North America (1 study, 4.17%), Europe (1 study, 4.17%) and both African and Asian countries (1 study, 4.17%). Most of the included studies used only microscopy for identification of malaria parasites (16 studies, 66.7%). Most of the included studies used enzyme-linked immunosorbent assay (ELISA) to measure IFN-γ in the blood of participants (15 studies, 62.5%). Other characteristics of the included studies are shown in Table 1. In addition, details of the included studies are provided in Table S2.

**Table 1:** Characteristics of the 24 included studies.

Characteristics	N	Percentage (%, total = 24)
**Publication years**		
1994–2000	3	12.5
2000–2010	10	41.7
2011–2017	11	45.8
**Study designs**		
Case–control studies	10	41.7
Prospective observational studies	9	37.5
Cross-sectional study	3	12.5
Prospective cohort studies	2	8.33
**Study locations**		
Africa	11	45.8
Asia	7	29.2
South America	3	12.5
North America	1	4.17
Europe	1	4.17
Africa and Asia	1	4.17
**Age groups of participants**		
Children	10	41.7
Adults	7	29.2
All age groups	7	29.1
**Malaria detection methods**		
Microscopy	16	66.7
Microscopy/PCR	3	12.5
Microscopy/RDT	2	8.33
Microscopy/PCR/IFA	1	4.17
PCR	1	4.17
Not specified	1	4.17
**TNF-α measurement**		
ELISA	15	62.5
Bead-based assays	9	37.5

### Quality of the included studies

The quality of the included studies was determined using the STROBE checklist. The assessment results showed that seven of nine prospective observational studies (77.8%) were of high quality, while three were of moderate quality (22.2%). Eight of the ten case–control studies (70%) were of high quality, while two were of moderate quality (30%). Two prospective cohort studies were of high quality, and one cross-sectional study was of moderate quality (Table S3).

### Qualitative synthesis

The difference in IFN-γ levels between patients with severe and uncomplicated malaria was qualitatively described using the results from individual studies. Overall, nine studies (37.5%) reported that IFN-γ levels were significantly higher in patients with severe malaria than those in patients with uncomplicated malaria^18,19,23–25,36–39^. Meanwhile, 14 studies (58.3%) reported no statistically significant differences in IFN-γ levels between patients with severe malaria and those with uncomplicated malaria^17,20,22,40–51^. Only one study (4.17%) reported that IFN-γ levels were significantly lower in patients with severe malaria than those in patients with uncomplicated malaria (Table 2). Among studies that reported significantly higher IFN-γ levels in patients with severe malaria than in those with uncomplicated malaria^18,19,23–25,36–39^, six studies (66.7%) reported severe *P. falciparum* infections^18,19,23,25,38,39^ and three studies (33.3%) reported severe *P. vivax* infections^24,36,37^. Meanwhile, 14 studies reported no difference in IFN-γ levels between the two groups^17,20,22,40–51^, and one study that reported significantly lower IFN-γ levels in patients with severe malaria than in those with uncomplicated malaria^17^ reported only *P. falciparum* infection.

**Table 2:** Differences in IFN-γ levels between patients with severe and uncomplicated malaria based on qualitative data.

Studies	*Plasmodium* species	IFN-γ levels*
Andrade et al.^36^	*P. vivax*	Significantly higher
Mirghani et al.^23^	*P. falciparum*	Significantly higher
Munde et al.^38^	*P. falciparum*	Significantly higher
Singotamu et al.^24^	*P. vivax*	Significantly higher
Tangteerawatana et al.^39^	*P. falciparum*	Significantly higher
Wroczyńska et al.^25^	Severe *P. falciparum* vs. uncomplicated *P. falciparum*/*P. vivax*/*P. ovale*/*P. malariae*	Significantly higher
Lopera-Mesa et al.^18^	*P. falciparum*	Significant higher (cerebral malaria), no difference (noncerebral severe malaria)
Mandala et al.^19^	*P. falciparum*	Significantly higher (cerebral malaria), no difference (severe malarial anaemia)
Mendonça et al.^37^	*P. vivax*	Significantly higher (cerebral malaria)
Berg et al.^40^	*P. falciparum*	No significant difference
Duarte et al.(Gabon)^22^	*P. falciparum*	No significant difference
Duarte et al.,(India)^22^	*P. falciparum*	No significant difference
Ghanchi et al.^41^	*P. falciparum*	No significant difference
Jain et al.^42^	*P. falciparum*	No significant difference
Jakobsen et al.^43^	*P. falciparum*	No significant difference
Kwiatkowski et al.^44^	*P. falciparum*	No significant difference
Nmorsi et al.^45^	*P. falciparum*	No significant difference
Ong’echa et al.^20^	*P. falciparum*	No significant difference
Perera et al.^48^	*P. falciparum*	No significant difference
Phawong et al.^47^	*P. falciparum*	No significant difference
Prakash et al.^48^	*P. falciparum*	No significant difference
Rovira-Vallbona et al.^49^	*P. falciparum*	No significant difference
Sinha et al.^52^	*P. falciparum*	No significant difference
Yamada-Tanaka et al.^51^	*P. falciparum*	No significant difference
Oyegue-Liabagui et al.^17^	*P. falciparum*	Significantly lower

*Results based on the statistical tests by included studies.

### Quantitative synthesis (meta-analysis)

**Figure 2:**
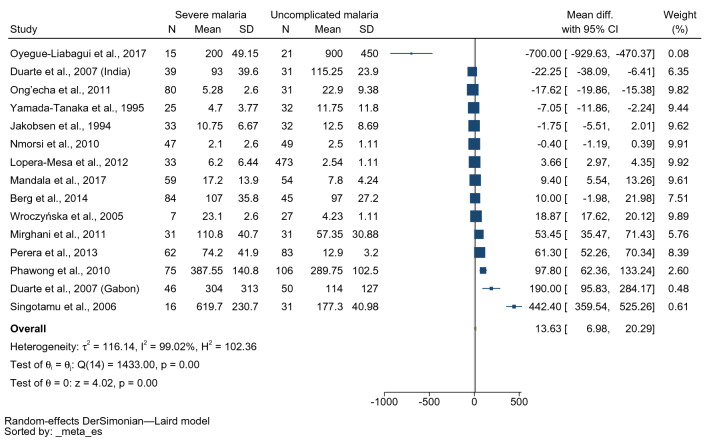
Forest plot demonstrating the pooled MD of INF-γ levels between patients with severe and uncomplicated malaria. *CI* confidence interval, *SD* standard deviation.

The difference in mean IFN-γ levels between patients with severe and uncomplicated malaria was estimated using 15 studies^17–20,22–25,40,43,45–47,51^. The lowest MD was identified in the study conducted in Gabonese children (− 700 pg/mL, 95% confidence interval [CI] − 929.63–470.37 pg/mL)^17^. The highest MD was identified in the study conducted in India (442.40 pg/mL, 95% CI 359.54–552.26 pg/mL)^24^. Four studies demonstrated that patients with severe malaria had lower mean IFN-γ levels than those with uncomplicated malaria^17,20,22,51^. Two studies demonstrated no difference in mean IFN-γ levels between patients with severe malaria and those with uncomplicated malaria^43,45^. Meanwhile, six studies showed that patients with severe malaria had higher mean IFN-γ levels than those with uncomplicated malaria^22–25,46,47^. Overall, the results demonstrated that patients with severe malaria had higher mean IFN-γ levels than those with uncomplicated malaria (p < 0.001, MD: 13.63 pg/mL, 95% CI 6.98–20.29 pg/mL, I^2^: 99.02%, 14 studies/15 study sites, 652 severe cases/1096 uncomplicated cases, Fig. 2).

Because of the high heterogeneity of the effect estimates among the included studies, a meta-regression analysis incorporating study design, location (continent), age group and technique used to measure the IFN-γ levels was conducted to identify whether these covariates were the source(s) of heterogeneity. The findings showed that study design (*p* < 0.001), age group (*p* = 0.001), location (continent, *p* < 0.001) and technique used to measure the IFN-γ levels (*p* < 0.009) were sources of heterogeneity in the effect estimates among the included studies. Therefore, subgroup analyses of study design, age group, location (continent) and technique used to measure the IFN-γ levels were performed.

**Figure 3:**
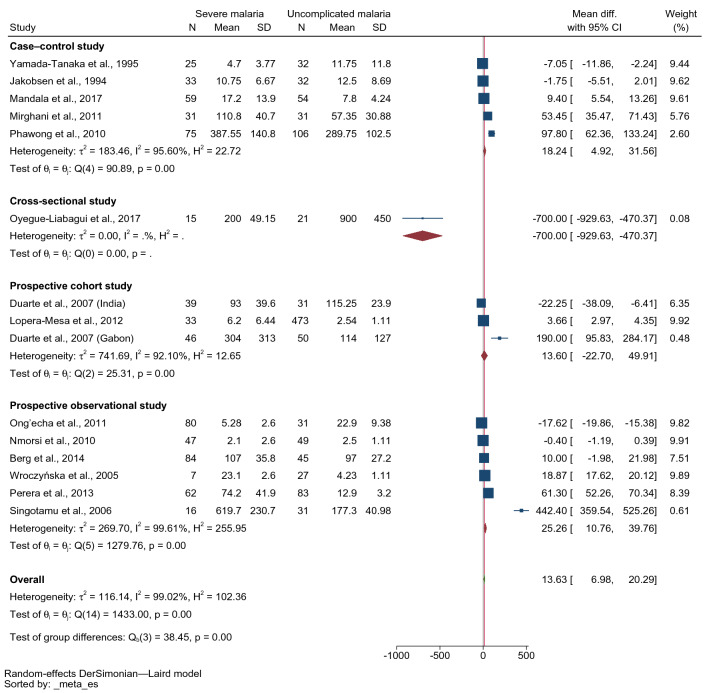
Forest plot demonstrating the pooled MD of INF-γ levels between patients with severe and uncomplicated malaria stratified by study design**.** *CI* confidence interval, *SD* standard deviation.

The subgroup analysis by the study design showed that higher mean IFN-γ levels were found in patients with severe malaria than in those with uncomplicated malaria in prospective observational studies (MD: 25.26 pg/mL, 95% CI 10.76–39.76 pg/mL, I^2^: 99.61%, six studies, 296 severe cases/266 uncomplicated cases) and case–control studies (MD: 18.24 pg/mL, 95% CI 4.92–31.56 pg/mL, I^2^: 95.6%, five studies, 223 severe cases/255 uncomplicated cases). Meanwhile, no difference in mean IFN-γ levels between patients with severe and those with uncomplicated malaria was identified in prospective cohort studies (MD 13.6 pg/mL, 95% CI − 22.7–49.91 pg/mL, I^2^: 92.1%, two studies with three study sites, 118 severe cases/554 uncomplicated cases, Fig. 3).

**Figure 4:**
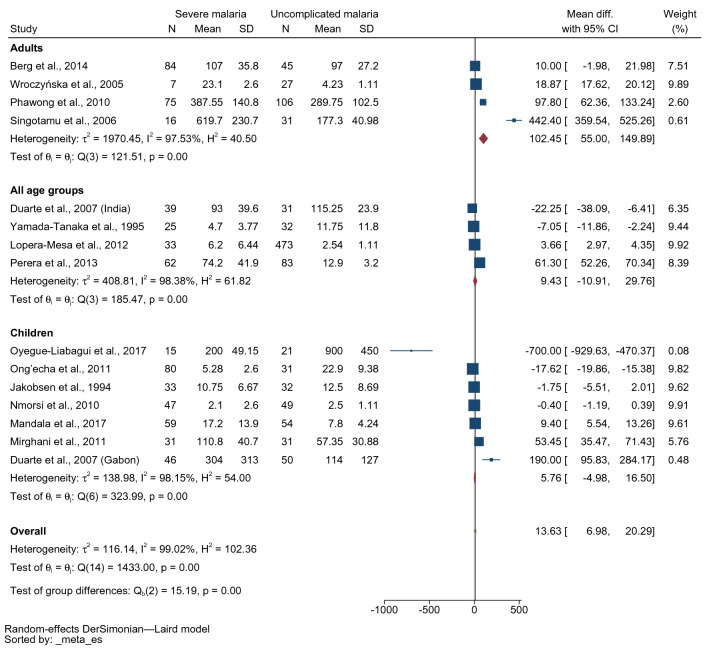
Forest plot demonstrating the pooled MD of INF-γ levels between patients with severe and uncomplicated malaria stratified by age group**.** *CI* confidence interval, *SD* standard deviation.

The subgroup analysis by the age of the enrolled patients showed no differences in mean IFN-γ levels in patients with severe malaria and those with uncomplicated malaria in children (MD 5.76 pg/mL, 95% CI − 4.98–16.5 pg/mL, I^2^: 98.15%, seven studies, 311 severe cases/268 uncomplicated cases) and all age groups (MD 9.43 pg/mL, 95% CI − 10.91–29.76 pg/mL, I^2^: 98.38%, four studies, 159 severe cases/619 uncomplicated cases). Meanwhile, higher mean IFN-γ levels were found in adults with severe malaria than in those with uncomplicated malaria (MD 102.45 pg/mL, 95% CI 55–149.89 pg/mL, I^2^: 97.53%, four studies, 182 severe cases/209 uncomplicated cases, Fig. 4).

**Figure 5:**
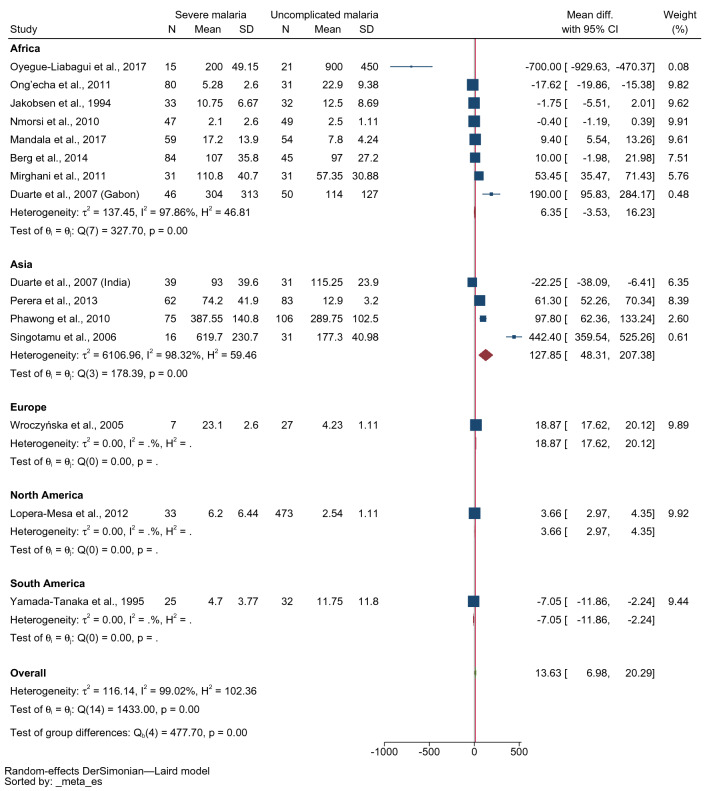
Forest plot demonstrating the pooled MD of INF-γ levels between patients with severe and uncomplicated malaria stratified by location (continent). *CI* confidence interval, *SD* standard deviation.

The subgroup analysis by the study location (continent) showed higher mean IFN-γ levels in patients with severe malaria than in those with uncomplicated malaria among studies that were conducted in Asia (MD: 127.85 pg/mL, 95% CI 48.31–207.38 pg/mL, I^2^: 98.32%, four studies, 192 severe cases/251 uncomplicated cases). However, no differences in mean IFN-γ levels between patients with severe and those with uncomplicated malaria were found among studies conducted in Africa (MD: 6.35 pg/mL, 95% CI − 3.53–16.23 pg/mL, I^2^: 97.86%, eight studies, 395 severe cases/313 uncomplicated cases, Fig. 5). Among studies conducted in Africa, there were no differences in mean IFN-γ levels in children with severe malaria and those with uncomplicated malaria among studies that enrolled children (MD: 5.76 pg/mL, 95% CI − 4.98–16.50 pg/mL, I^2^: 98.15%, seven studies, 311 severe cases/268 uncomplicated cases, Supplementary Fig. S1). Among studies conducted in Asia, no difference in mean IFN-γ levels between patients with severe malaria and those with uncomplicated was evident in adults (MD 267.98 pg/mL, 95% CI − 69.7–605.66 pg/mL, I^2^: 98.15%, two studies, 91 severe cases/137 uncomplicated cases) and all age groups (MD 17.79 pg/mL, 95% CI − 62.09–101.66 pg/mL, I^2^: 98.76%, two studies, 101 severe cases/114 uncomplicated cases, Supplementary Fig. S2).

**Figure 6:**
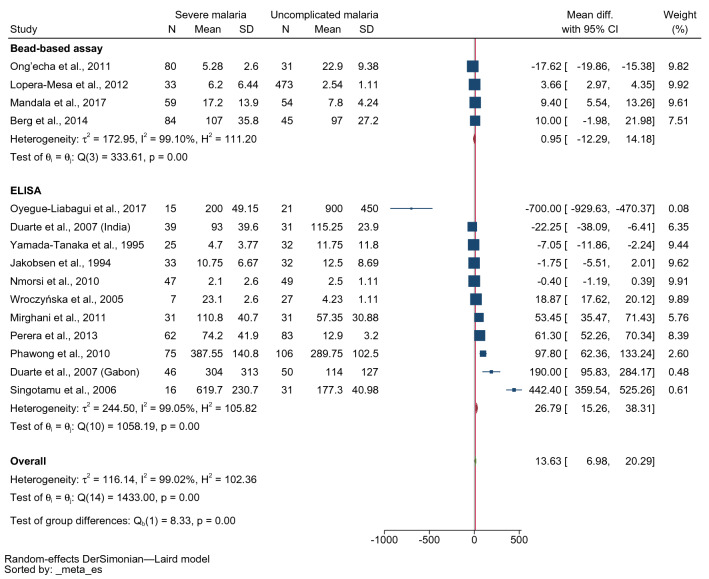
Forest plot demonstrating the pooled MD of INF-γ levels between patients with severe and uncomplicated malaria stratified by methods for INF-γ measurement. *CI* confidence interval; SD, standard deviation.

The subgroup analysis by the technique for IFN-γ measurement showed higher mean IFN-γ levels in patients with severe malaria than in those with uncomplicated malaria among studies using ELISA for IFN-γ measurement (MD: 26.79 pg/mL, 95% CI 15.26–38.31 pg/mL, I^2^: 99.05%, ten studies with 11 study sites). However, no differences in mean IFN-γ levels between patients with severe and those with uncomplicated malaria were found among studies using bead-based assays for IFN-γ measurement (MD: 0.95 pg/mL, 95% CI − 12.29–14.18 pg/mL, I^2^: 99.10%, four studies, 256 severe cases/603 uncomplicated cases, 396 severe cases/492 uncomplicated cases, Fig. 6).

### Sensitivity analysis

**Figure 7:**
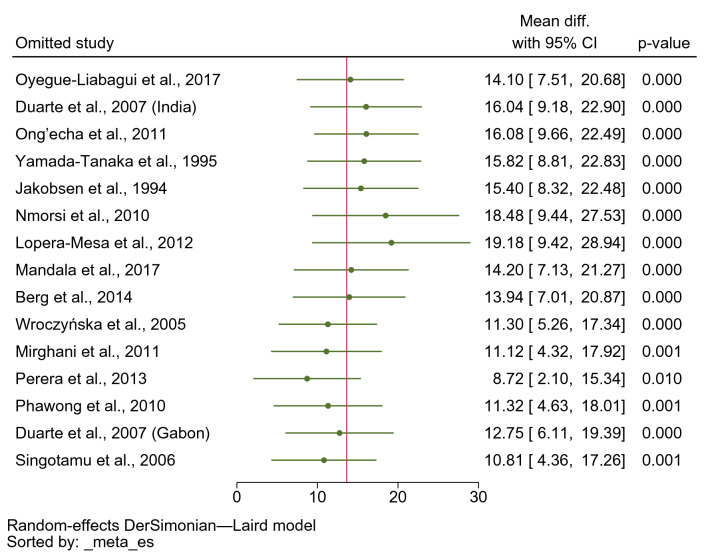
Forest plot demonstrating the sensitivity analysis (leave-one-out method) of the pooled MD of INF-γ levels between patients with severe and uncomplicated malaria**.** *CI* confidence interval.

A sensitivity analysis was conducted to test whether the meta-analysis results were robust. This inquiry into the sensitivity of the meta-analysis of IFN-γ levels in severe and uncomplicated malaria demonstrated that patients with severe malaria had higher mean IFN-γ levels than those with uncomplicated malaria when the leave-one-out method was applied (p < 0.05, Fig. 7), indicating that the results of the meta-analysis were robust. The sensitivity analysis between studies that reported mean/standard deviation and median/range and studies that reported mean without standard deviation was performed. Results showed that patients with severe malaria had higher mean IFN-γ levels than those with uncomplicated malaria (p < 0.001, MD: 20.12 pg/mL, 95% CI 9.56–30.69 pg/mL, I^2^: 98.39%, 12 studies/13 study sites, 598 severe cases/1020 uncomplicated cases, Supplementary Fig. S3). The sensitivity analysis between studies that reported mean/standard deviation and those with median/range of IFN-γ levels in patients with severe and uncomplicated malaria was conducted using the subgroup analysis. Results indicated that patients with severe malaria had higher mean IFN-γ levels than those with uncomplicated malaria among studies that reported mean/standard deviation of IFN-γ levels (MD: 38.64 pg/mL, 95% CI 20.53–56.75 pg/mL, I^2^: 99.57%, five studies, 147 severe cases/211 uncomplicated cases). Meanwhile, no differences in mean IFN-γ levels between patients with severe and those with uncomplicated malaria were found among studies that reported median/range of IFN-γ levels (MD: 7.74 pg/mL, 95% CI − 1.24–16.71 pg/mL, I^2^: 97.93%, nine studies with ten study sites, 505 severe cases/885 uncomplicated cases, Supplementary Fig. S4).

### Publication bias

**Figure 8:**
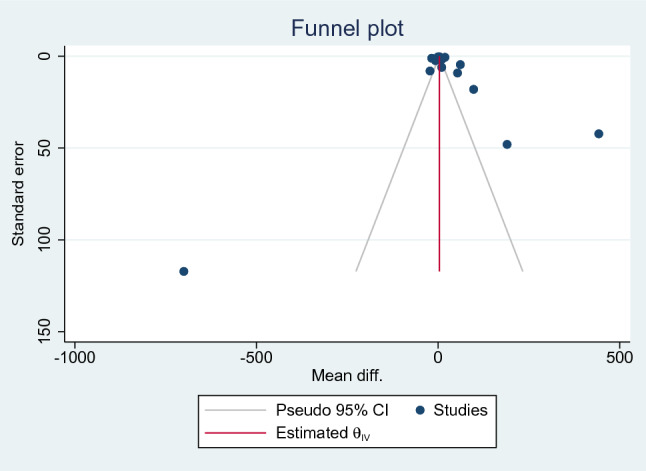
Funnel plot of the studies included in the meta-analysis between patients with severe malaria and uncomplicated malaria. *CI* confidence interval.

The publication bias of the effect estimates among the included studies was assessed by visualisation of funnel plot symmetry and Egger’s test for small-study effect. In the meta-analysis between IFN-γ levels between patients with severe and those with uncomplicated malaria, asymmetry of the funnel plot was suspected (Fig. 8), and Egger’s test exhibited a small-study effect (*p* < 0.001), showing that publication bias was discovered. The trim-and-fill method was applied to adjust the pooled effect estimate, and the rresults showed that the pooled MD of IFN-γ levels between patients with severe and uncomplicated malaria after adjusting for publication bias was 13.634 pg/mL (95% CI 6.979–20.29 pg/mL).

## Discussion

The main feature of this study was the comparison of IFN-γ levels between patients with severe and those with uncomplicated malaria. The results of the qualitative synthesis demonstrated that most studies that investigated IFN-γ levels in both patients with severe and uncomplicated malaria revealed no statistically significant IFN-γ levels between the two clinical outcomes. Meanwhile, few studies have reported that IFN-γ levels were significantly higher in patients with severe malaria than those in patients with uncomplicated malaria. For quantitative synthesis by meta-analysis, the higher IFN-γ levels were observed in patients with severe malaria than those in patients with uncomplicated malaria. Although a high degree of heterogeneity of the outcome existed, the meta-analysis results implied that IFN-γ levels were associated with malaria severity, i.e. increased levels positively correlated with increased severity.

IFN-γ levels related to malaria severity were observed by in vitro studies^52–54^. Nevertheless, a study on children with malaria argued that reduced IFN-γ levels were associated with malaria severity^55^. A discrepancy in IFN-γ levels and malaria severity between studies might be because of differences in the different participants enrolled in each as suggested previously^20^. Various severe complications among patients, such as anaemia, parasitemia levels or cerebral malaria, might cause the differences in the MD in IFN-γ levels between severe and uncomplicated malaria. A previous study indicated that IFN-γ production was associated with the reduced prevalence of anaemia caused by *P. falciparum*; hence, IFN-γ was suggested to be an immunity-based protection against severe malarial anaemia^17^. A previous study found that IFN-γ levels were negatively associated with parasitemia, suggesting that this cytokine has antiparasitic effects^46^. Increased IFN-γ levels were also related to high malarial parasitemia^52^. This association may be due to malarial parasitemia caused by the production of IFN-γ by immune cells^56^. In comparison to severe malarial anaemia, patients with cerebral malaria showed higher levels of pro-inflammatory/Th1 cytokines^19^, indicating that the production of pro-inflammatory cytokines in severe malaria is poorly regulated^21^.

Although the meta-analysis results exhibited higher mean IFN-γ levels in severe malaria than in uncomplicated malaria, the degree of heterogeneity among studies included in the meta-analysis was extremely high. The meta-regression and subgroup analyses of study design, continents, age groups, and techniques for IFN-γ measurement proposed that these parameters were sources of heterogeneity in the outcome. Considering study design as a source of heterogeneity, prospective observational and case–control studies showed higher mean IFN-γ levels were found in patients with severe malaria compared to those with uncomplicated malaria. Meanwhile, the prospective cohort studies indicated no difference. These results might be because only two prospective cohort studies were included in the subgroup analysis, which might bias the results of the subgroup analysis. Considering the continent as another source of heterogeneity, studies conducted in Asia showed higher mean IFN-γ levels in patients with severe malaria than those with uncomplicated malaria. However, there was no difference in mean IFN-γ levels among studies performed in Africa, indicating that the different populations investigated may have had various immune responses to malaria. In Africa, where falciparum malaria is endemic, the populations are more exposed to infections; hence, they may have acquired immune responses against malaria infections or severity^57–60^. Therefore, it is possible that both patients with severe and uncomplicated malaria showed comparable cytokine responses, which causes non-statistical significance between groups in this meta-analysis. In Asia, where falciparum malaria is less endemic, most populations are less exposed to infections; hence, they are non-immune or semi-immune responses to malaria infections^61^. Therefore, patients with severe malaria in Asian countries might develop a stronger immune response to the infections than those with uncomplicated malaria. For example, Singotamu et al.^24^ in India indicated that *P. vivax* infections demonstrated very high mean IFN-γ levels (619.7 pg/mL) in patients with severe malaria compared with uncomplicated malaria (177.3 pg/mL). Additional background histories or co-occurrence with pathogens other than malarial ones in various areas might manifest different immune responses in patients with malaria. The histories of the individuals studied may add a layer of complexity to the findings on the immune response to malaria^6^.

Considering age groups as another source of heterogeneity in the outcome, no differences in mean IFN-γ levels in children with severe malaria and those with uncomplicated malaria, but adults with severe malaria showed higher mean IFN-γ levels than those with uncomplicated malaria. This result might be explained by the fact that studies enrolling children with severe malaria were conducted in Africa, where malaria is endemic. Meanwhile, studies enrolling adults with severe malaria were conducted in Asia, where malaria is less endemic. Across sub-Saharan Africa, where the disease is hyper-endemic, most people are almost continuously infected with *P. falciparum*. Most infected adults rarely experience severe disease because of the acquired immunity against the infection. In areas where malaria is less endemic, such as Asia, a higher risk of severe disease is frequently observed among adults than children as adults develop a stronger immune response to the infection, but infants and children occasionally do not. This reason explained the possible cause of high cytokine response, including IFN-γ response to the infection.

Considering the techniques for measuring IFN-γ as another source of heterogeneity of the outcome, studies using ELISA exhibited higher mean IFN-γ levels in patients with severe malaria than in those with uncomplicated malaria. However, studies using bead-based assays indicated no differences in mean IFN-γ levels between patients with severe and those with uncomplicated malaria. Multiplex bead-based assays provide the means to simultaneously measure multiple proteins in a single reaction compared to ELISA, which measures a single protein in a cone reaction^62^. A study comparing the overall performance of the two methods for cytokine profiles demonstrated that the ELISA and bead-based assays yielded similar results^63^. Notably, ELISA was more sensitive in the low concentration range of the standard curve, whereas bead-based assays could detect higher protein concentrations^63^. Another study that measured IFN-γ levels using both techniques indicated comparable detection of plasma IFN-γ, IL-4, IL-10, and TNF-α, but the ELISA missed a cytokine such as IL-5^64^. Therefore, no study yet warrants the difference in performance between the two techniques for detecting IFN-γ in the blood. However, the results of the subgroup meta-analysis might guide further studies to examine the difference in the real performance of these techniques.

IFN-γ contributes to the activation and differentiation of B lymphocytes, T lymphocytes and macrophages^65–67^. The IFN-gamma receptor (R) locations were in lymphoid organs such as the B-cell areas of lymph nodes, spleen, tonsils, and in epithelial tissues of the intestinal system, lung, and endometrial mucosa cells^68^. Studies in mice models indicated that treatment of mice infected with blood-stage *P. berghei* by anti-IFN-γ antibody failed to control the infection parasites^69,70^. Additionally, delayed parasite elimination was found among IFN-γ-deficient or IFN-γ receptor (IFN-γR)-deficient mice or anti-IFN-γ antibody-treated mice^71–73^. During *Plasmodium* infection, γδ T-cells that express CD40 ligand produce IFN-γ in response to infection by enhancement of dendritic cell activation to remove malaria parasites^74^. In the pathogenesis of severe malaria, many studies have indicated that IFN-γ is vital for developing severe malaria, particularly cerebral malaria, by affecting endothelial integrity^70,72,75,76^. During cerebral malaria, IFN—producing CD8 + T-cells are recruited to the brain and cause cerebral pathology by destroying the blood–brain barrier in perforin- and granzyme-dependent manner^77,78^. IFN-γ production is modulated by several cytokines such as IL-12 and IL-18 or broadly reactive antigen receptors^79^. In the study by Wroczyńska et al.^25^, increased IFN-γ accompanied by increased IL-18 levels were observed in patients with severe malaria, indicating that excessive production of both cytokines is associated with severe malaria infections. IL-18-dependent IFN-γ overproduction was reported to relate to decreased IL-12 levels^25^. Therefore, these data suggested that severe malaria is associated with increased IFN-γ and decreased IL-12 levels, indicating the occurrence of immunoregulation in resolving malaria infection^25^. Furthermore, reduced IL-12 levels were associated with suppression of Th1 cytokine activation by NK cells or CD8 cells^56^. The previous studies also showed that IFN-γ was associated with IL-10 and IL-6, indicating a balance between these cytokines^23,80^. IFN-γ and IL-10 were markedly increased in patients with severe malaria^48,81^.

This study had some limitations. First, the degree of heterogeneity was extreme in the meta-analysis. Although meta-regression and subgroup analyses were conducted to identify the source(s) of heterogeneity, the heterogeneity remained in the subgroup analysis, showing that other factors confound the association between IFN-γ levels and malaria severity. Second, publication bias among the studies included in the meta-analysis was noted. Therefore, the pooled effect estimate (MD of IFN- γ levels) after applying the trim-and-fill method should be considered.

## Conclusion

In conclusion, patients with severe malaria present higher IFN-γ levels than those with uncomplicated malaria, although the heterogeneity of the outcomes is yet to be elucidated. To confirm whether alteration in IFN-γ levels of patients with malaria may indicate disease severity and/or poor prognosis, further studies are warranted.

## Supplementary Information

Supplementary Figure S1.

Supplementary Figure S2.

Supplementary Figure S3.

Supplementary Figure S4.

Supplementary Table S1.

Supplementary Table S2.

Supplementary Table S3.

## Data Availability

All data generated or analyzed during this study are included in this published article and its supplementary information files.
