# Author Correction: Increased interferon-γ levels and risk of severe malaria: a meta-analysis

**DOI:** 10.1038/s41598-022-25996-4

**Published:** 2022-12-09

**Authors:** Aongart Mahittikorn, Wanida Mala, Frederick Ramirez Masangkay, Kwuntida Uthaisar Kotepui, Polrat Wilairatana, Manas Kotepui

**Affiliations:** 1 Department of Protozoology, Faculty of Tropical Medicine, Mahidol University, Bangkok, Thailand; 2 Medical Technology, School of Allied Health Sciences, Walailak University, Tha Sala, Nakhon Si Thammarat Thailand; 3 Department of Medical Technology, Faculty of Pharmacy, University of Santo Tomas, Manila, Philippines; 4 Department of Clinical Tropical Medicine, Faculty of Tropical Medicine, Mahidol University, Bangkok, Thailand

Correction to: *Scientific Reports* 10.1038/s41598-022-21965-z, published online 07 November 2022

In the original version of this Article, the Acknowledgements section has been removed, as the funder has no role in the data collection, analysis, and interpretation of the data.

The original Article has been corrected.

