# Supplementary figures and images for: Increased interferon-γ levels and risk of severe malaria: a meta-analysis
Source: Sci Rep. 2022 Nov 7;12:18917. doi: 10.1038/s41598-022-21965-z (PMC9640646; doi:10.1038/s41598-022-21965-z)

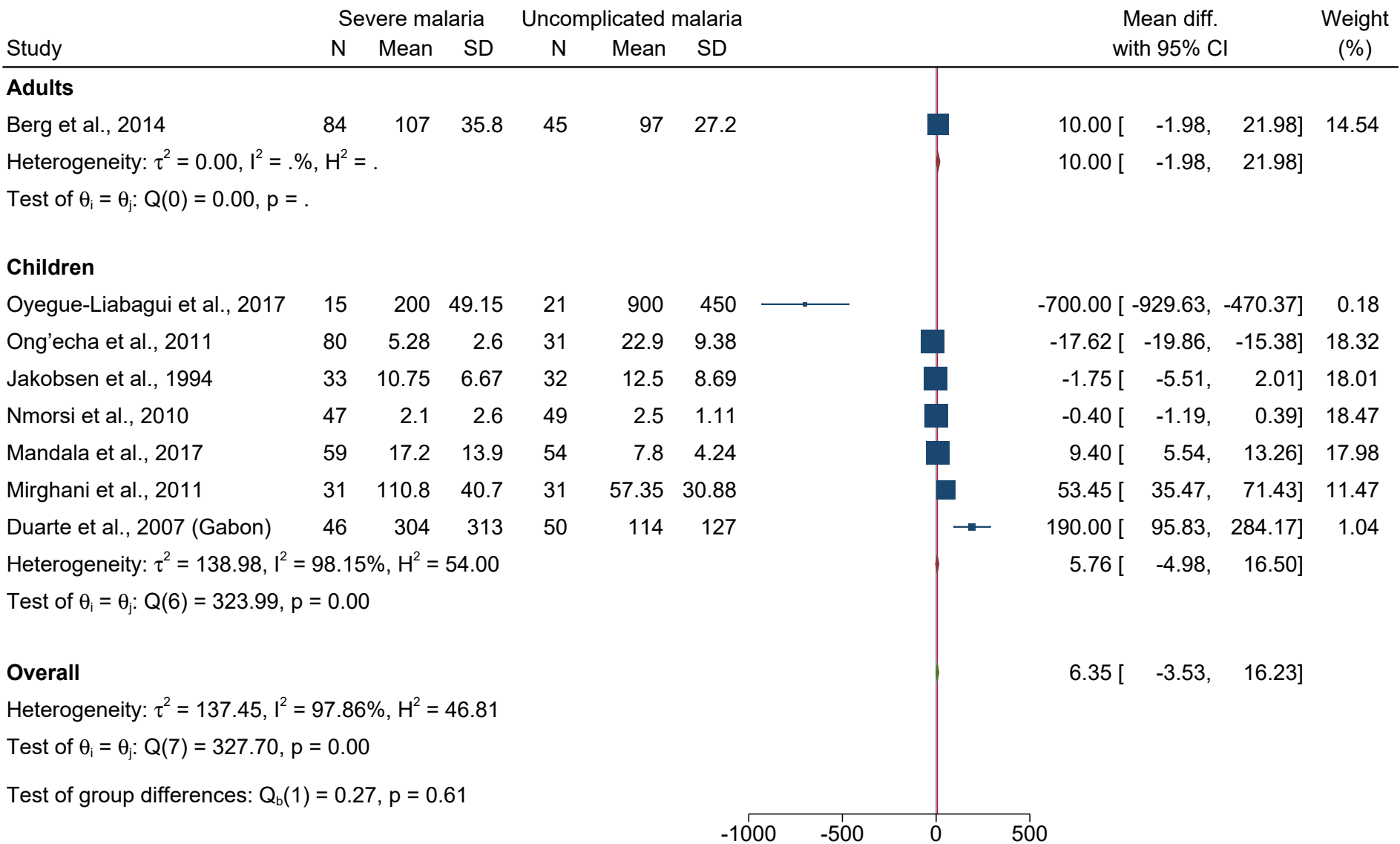

Supplement: Supplementary file 1 — Supplementary Figure S1. [file 41598_2022_21965_MOESM1_ESM.pdf]

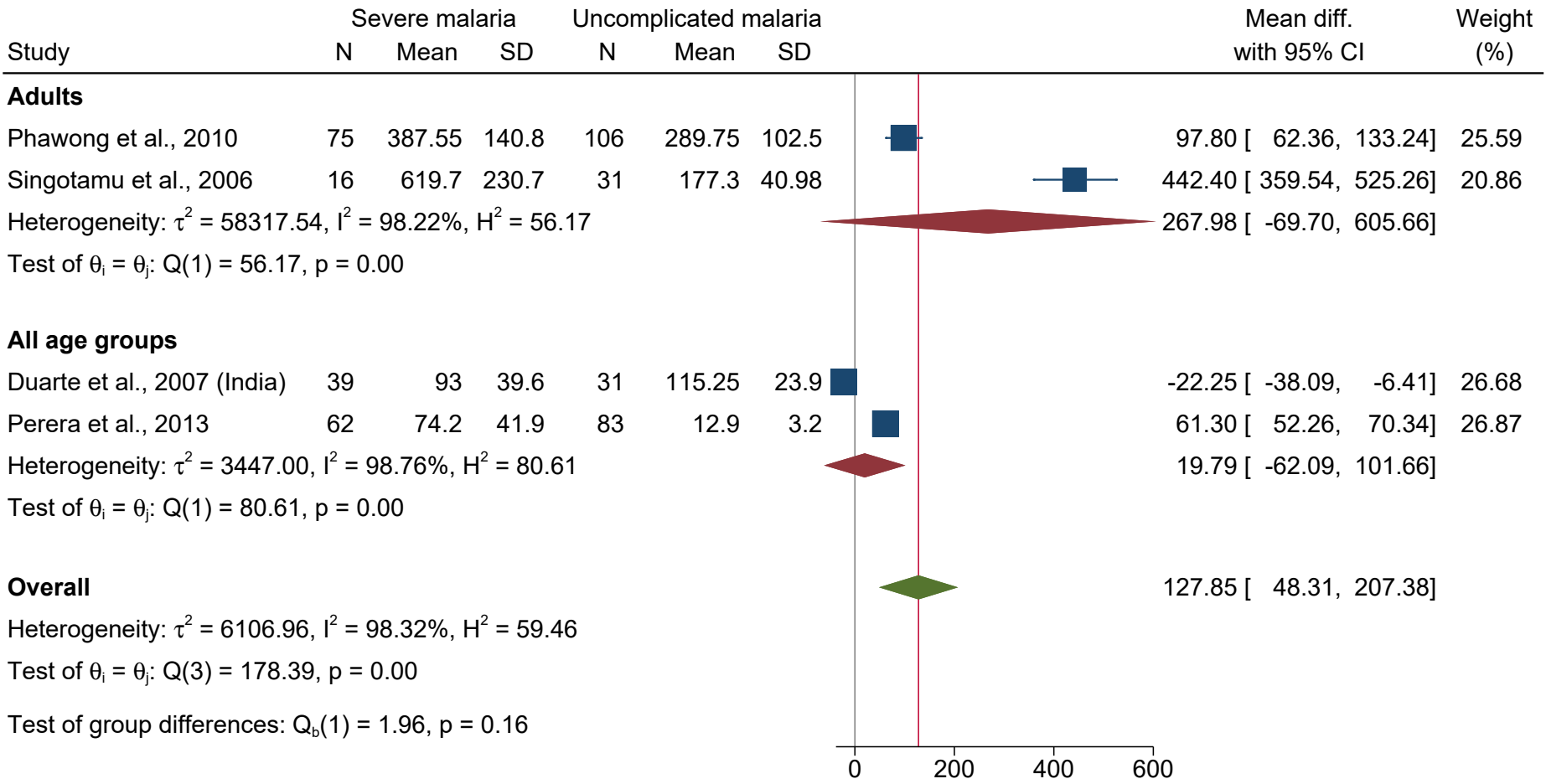

Supplement: Supplementary file 2 — Supplementary Figure S2. [file 41598_2022_21965_MOESM2_ESM.pdf]

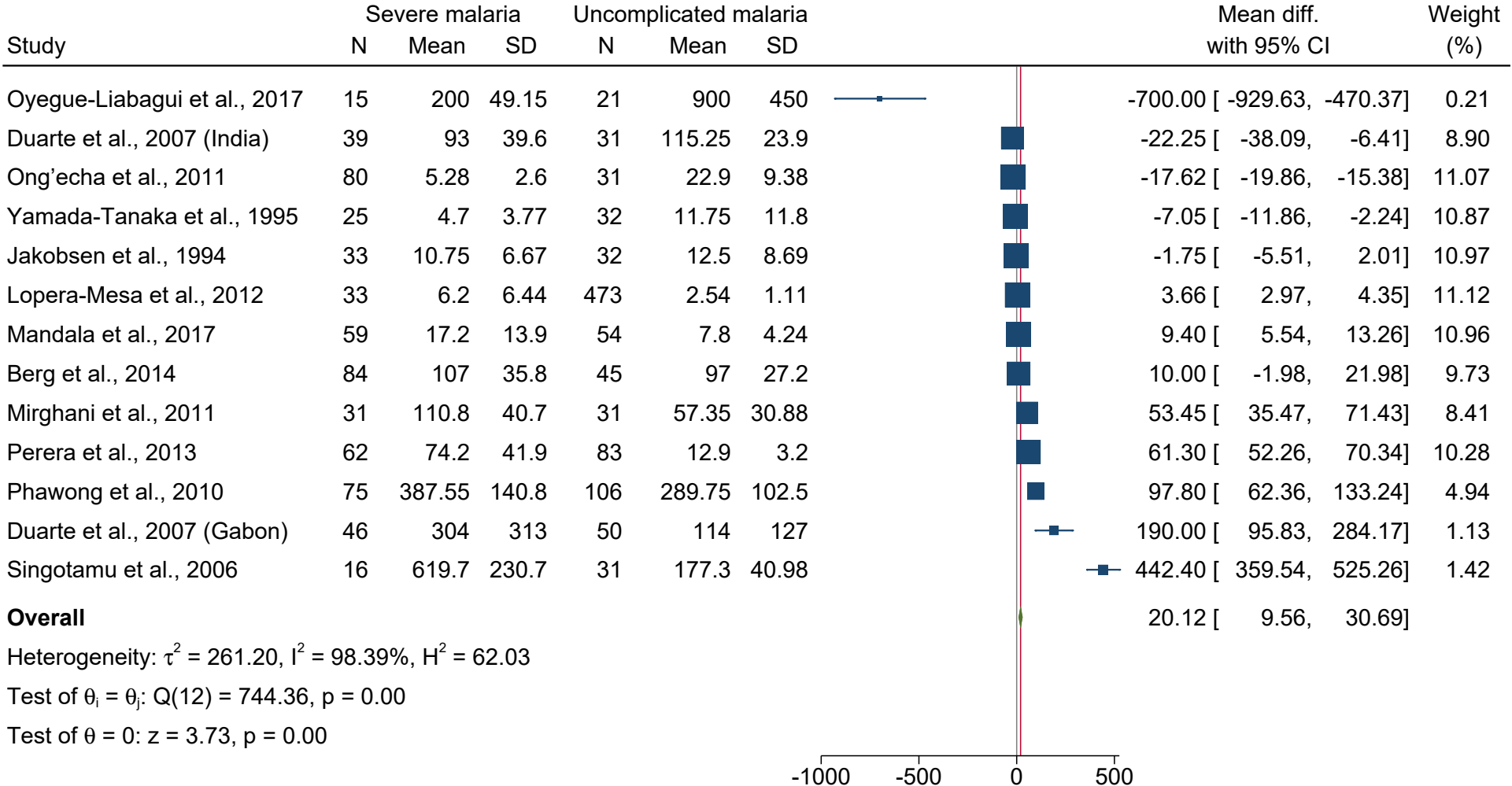

Supplement: Supplementary file 3 — Supplementary Figure S3. [file 41598_2022_21965_MOESM3_ESM.pdf]

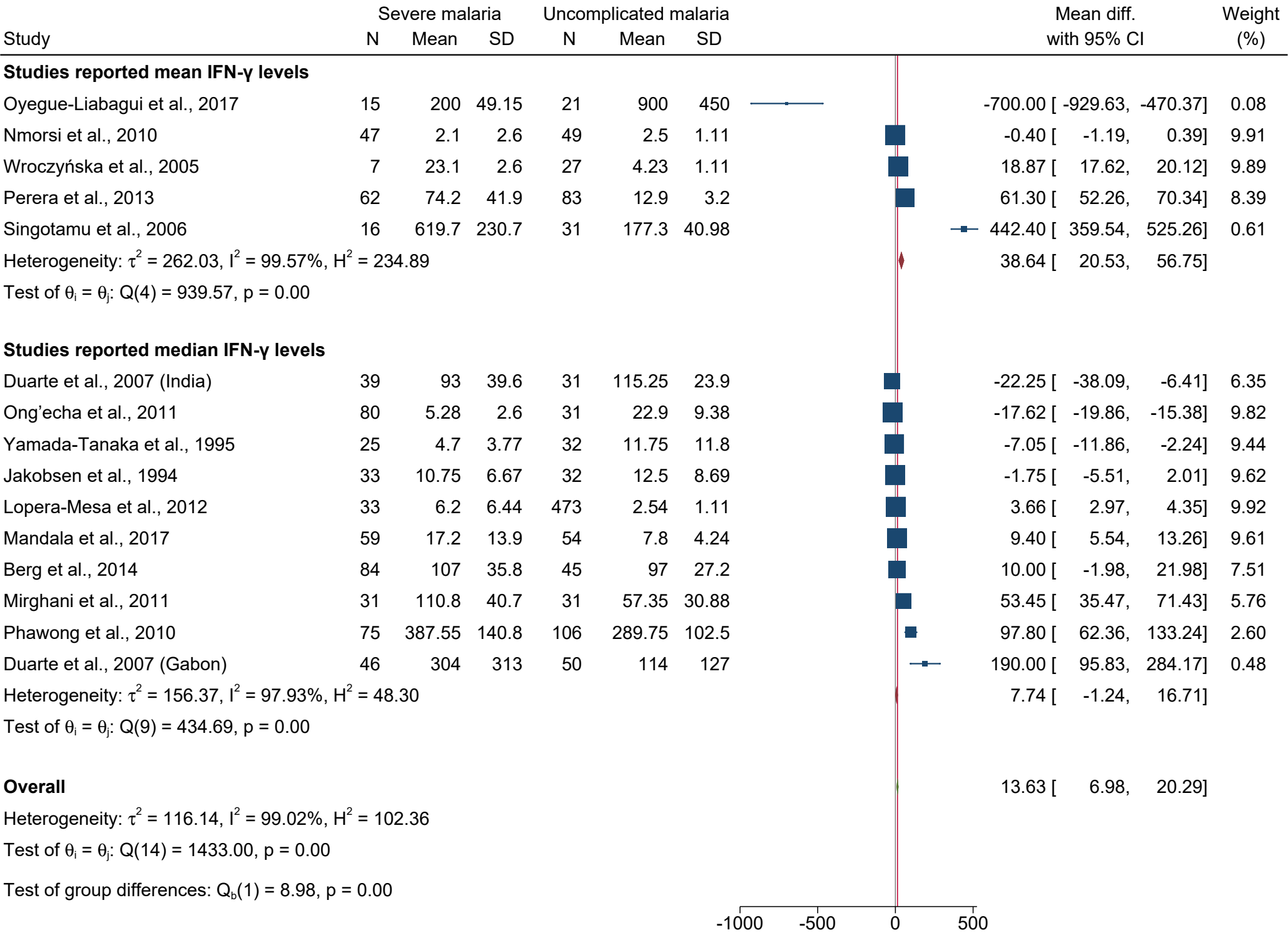

Supplement: Supplementary file 4 — Supplementary Figure S4. [file 41598_2022_21965_MOESM4_ESM.pdf]
