# Supplementary material for: Increased interferon-γ levels and risk of severe malaria: a meta-analysis
Source: Sci Rep. 2022 Nov 7;12:18917. doi: 10.1038/s41598-022-21965-z (PMC9640646; doi:10.1038/s41598-022-21965-z)
Supplement: Supplementary file 5 — Supplementary Table S1. [file 41598_2022_21965_MOESM5_ESM.docx]

**Increased interferon-γ levels and risk of severe malaria: a meta-analysis**

Aongart Mahittikorn^1^, Wanida Mala^2^, Frederick Ramirez Masangkay^3^, Kwuntida Uthaisar Kotepui^2^, Polrat Wilairatana^4^, Manas Kotepui^2*^

^1^ Department of Protozoology, Faculty of Tropical Medicine, Mahidol University, Bangkok, Thailand

^2^Medical Technology, School of Allied Health Sciences, Walailak University, Tha Sala, Nakhon Si Thammarat, Thailand

^3^Department of Medical Technology, Faculty of Pharmacy, University of Santo Tomas, Manila, Philippines.

^4^Department of Clinical Tropical Medicine, Faculty of Tropical Medicine, Mahidol University, Bangkok, Thailand

**^*^Corresponding author**

Manas Kotepui: [manas.ko@wu.ac.th](mailto:manas.ko@wu.ac.th), Tel.: +66954392469

Aongart Mahittikorn: aongart.mah@mahidol.ac.th

Wanida Mala: [wanida.ma@wu.ac.th](mailto:wanida.ma@wu.ac.th)

Frederick Ramirez Masangkay: frederick_masangkay2002@yahoo.com

Kwuntida Uthaisar Kotepui: [kwuntida.ut@wu.ac.th](mailto:kwuntida.ut@wu.ac.th)

Polrat Wilairatana: [polrat.wil@mahidol.ac.th](mailto:polrat.wil@mahidol.ac.th)

**Table S1. Search term**

| **Databases** | **Search terms/Search strategy** | **Date** |
| --- | --- | --- |
| PubMed | (interferon OR IFN OR interferon-gamma OR interferon-g OR IFN-g OR interferon-γ OR IFN-γ) AND (severe OR complicated) AND (malaria OR plasmodium)  Search results: 524 | 10 July 2022 |
| Scopus | (interferon OR IFN OR interferon-gamma OR interferon-g OR IFN-g OR interferon-γ OR IFN-γ) AND (severe OR complicated) AND (malaria OR plasmodium)  Search option: Title, abstract, keywords  Search results: 558 | 10 July 2022 |
| Embase | (interferon OR IFN OR interferon-gamma OR interferon-g OR IFN-g OR interferon-γ OR IFN-γ) AND (severe OR complicated) AND (malaria OR plasmodium)  Search results: 792 | 10 July 2022 |
| MEDLINE | (interferon OR IFN OR interferon-gamma OR interferon-g OR IFN-g OR interferon-γ OR IFN-γ) AND (severe OR complicated) AND (malaria OR plasmodium)  Search results: 325 | 10 July 2022 |
| Web of Science | (interferon OR IFN OR interferon-gamma OR interferon-g OR IFN-g OR interferon-γ OR IFN-γ) AND (severe OR complicated) AND (malaria OR plasmodium)  Search option: All fields  Search results: 469 | 10 July 2022 |
