# Supplementary material for: Increased interferon-γ levels and risk of severe malaria: a meta-analysis
Source: Sci Rep. 2022 Nov 7;12:18917. doi: 10.1038/s41598-022-21965-z (PMC9640646; doi:10.1038/s41598-022-21965-z)
Supplement: Supplementary file 7 — Supplementary Table S3. [file 41598_2022_21965_MOESM7_ESM.docx]

**Increased interferon-gamma levels and risk of severe malaria: A meta-analysis**

Aongart Mahittikorn^1^, Wanida Mala^2^, Frederick Ramirez Masangkay^3^, Kwuntida Uthaisar Kotepui^2^, Polrat Wilairatana^4^, Manas Kotepui^2*^

^1^ Department of Protozoology, Faculty of Tropical Medicine, Mahidol University, Bangkok, Thailand

^2^Medical Technology, School of Allied Health Sciences, Walailak University, Tha Sala, Nakhon Si Thammarat, Thailand

^3^Department of Medical Technology, Faculty of Pharmacy, University of Santo Tomas, Manila, Philippines.

^4^Department of Clinical Tropical Medicine, Faculty of Tropical Medicine, Mahidol University, Bangkok, Thailand

**^*^Corresponding author**

Manas Kotepui: [manas.ko@wu.ac.th](mailto:manas.ko@wu.ac.th), Tel.: +66954392469

Aongart Mahittikorn: aongart.mah@mahidol.ac.th

Wanida Mala: [wanida.ma@wu.ac.th](mailto:wanida.ma@wu.ac.th)

Frederick Ramirez Masangkay: frederick_masangkay2002@yahoo.com

Kwuntida Uthaisar Kotepui: [kwuntida.ut@wu.ac.th](mailto:kwuntida.ut@wu.ac.th)

Polrat Wilairatana: [polrat.wil@mahidol.ac.th](mailto:polrat.wil@mahidol.ac.th)

**Table S3. Quality of the included studies**

**Prospective observational study**

|  | **Study** | **Score (out of 22)** | **Score (percentage)** | **Quality** |
| --- | --- | --- | --- | --- |
| 1. | Berg et al., 2014 | 21 | 95 | High |
| 2. | Mendonça et al., 2015 | 19 | 86 | High |
| 3. | Munde et al., 2012 | 19 | 86 | High |
| 4. | Nmorsi et al., 2010 | 16 | 73 | Moderate |
| 5. | Ong’echa et al., 2011 | 18 | 82 | High |
| 6. | Perera et al., 2013 | 18 | 82 | High |
| 7. | Singotamu et al., 2006 | 16 | 73 | Moderate |
| 8. | Tangteerawatana et al., 2007 | 19 | 86 | High |
| 9. | Wroczyńska et al., 2005 | 17 | 77 | High |

**Case-control study**

|  | **Study** | **Score (out of 22)** | **Score (percentage)** | **Quality** |
| --- | --- | --- | --- | --- |
| 1. | Jain et al., 2008 | 18 | 82 | High |
| 2. | Jakobsen et al., 1994 | 16 | 73 | Moderate |
| 3. | Kwiatkowski et al. 1990 | 16 | 73 | Moderate |
| 4. | Mandala et al., 2017 | 21 | 95 | High |
| 5. | Mirghani et al., 2011 | 17 | 77 | High |
| 6. | Phawong et al., 2010 | 19 | 86 | High |
| 7. | Prakash et al., 2006 | 20 | 91 | High |
| 8. | Rovira-Vallbona et al. 2012 | 21 | 95 | High |
| 9. | Sinha et al., 2010 | 19 | 86 | High |
| 10. | Yamada-Tanaka et al., 1995 | 18 | 82 | High |

**Prospective cohort study**

|  | **Study** | **Score (out of 22)** | **Score (percentage)** | **Quality** |
| --- | --- | --- | --- | --- |
| 1. | Duarte et al., 2007 | 19 | 86 | High |
| 2. | Lopera-Mesa et al., 2012 | 22 | 100 | High |

**Cross-sectional study**

|  | **Study** | **Score (out of 22)** | **Score (percentage)** | **Quality** |
| --- | --- | --- | --- | --- |
| 1. | Oyegue-Liabagui et al., 2017 | 16 | 73 | Moderate |

STROBE: Strengthening the Reporting of Observational Studies in Epidemiology
